# Association between maternal smoking during pregnancy and smoking behaviors in adult offspring

**DOI:** 10.3389/fpubh.2025.1505418

**Published:** 2025-03-28

**Authors:** Lin Yang, Jing Li, Yinzi Chen, Yanling Wei, Congying Song, Jingwen Zhang, Liang Dai, Yu Shi, Zuying Xiong, Ying Shan, Xiaoyan Huang

**Affiliations:** ^1^Health Management Center, Peking University Shenzhen Hospital, Peking University, Shenzhen, China; ^2^Renal Division, Peking University Shenzhen Hospital, Peking University, Shenzhen, China; ^3^Clinical Research Academy, Peking University Shenzhen Hospital, Peking University, Shenzhen, China; ^4^School of Public Health, Fudan University, Shanghai, China; ^5^Department of Ultrasound, Peking University Shenzhen Hospital, Peking University, Shenzhen, China

**Keywords:** maternal smoking, pregnancy, offspring, smoking, UK Biobank

## Abstract

**Background:**

Maternal smoking during pregnancy (MSP) is associated with offspring smoking. However, there is still scant evidence to support the association between MSP and smoking behaviors in adult offspring.

**Methods:**

This is a prospective cohort study based on the UK Biobank. Maternal smoking around birth was reported by the offspring through a questionnaire. Participants with unknown maternal smoking status were classified as having missing values. Logistic regression, linear regression and negative binomial regression models were used to estimate the associations of exposure to maternal smoking around birth with four outcomes of offspring smoking behaviors, including smoking status, age started smoking, pack years of smoking, and number of unsuccessful stop-smoking attempts.

**Results:**

We included 156,604, 101,204, 96,466, and 70,308 participants in the analyses of smoking status, age started smoking, pack years of smoking, and number of unsuccessful stop-smoking attempts, respectively. After adjusted for potential confounders, MSP demonstrated significant associations with offspring smoking (OR: 1.08 [95% CI: 1.07, 1.10]), age started smoking (beta per year: −0.83 [95% CI: −0.89, −0.77]), pack years of smoking (beta per pack-year: 3.51 [95% CI: 3.28, 3.74]) and number of unsuccessful stop-smoking attempts (IRR: 1.11 [95% CI 1.09, 1.13]). Subgroup analysis showed the excessive risks for smoking status and age started smoking in females, pack years of smoking in males, and for number of unsuccessful stop-smoking attempts in the non-breastfeeding group.

**Conclusion:**

The unfavorable effects of maternal smoking around birth might reach at least up to offspring’s middle even older age. Smoking cessation before pregnancy should be encouraged to prevent the transmission to the next generation.

## 1 Introduction

Maternal smoking during pregnancy (MSP) is a well-documented risk factor for a range of adverse outcomes in offspring, such as fetal-related, psychical, and behavioral problems (1–4). Despite of strengthening of tobacco control initiatives, MSP is still common worldwide and remains a public health challenge (5, 6). A systematic review study reported the global prevalence of ever and current cigarette smoking in women from 2010 to 2020 was 28 and 17%, respectively (5).

More and more animal and epidemiologic studies have proved the association between MSP and offspring smoking (7–17). Most studies focus on the adolescence, a developmental period during which smoking behaviors are not stabilized maturely (8, 9, 11, 12, 14, 16–19). However, there have been only a few studies targeting the adults and presenting conflicting results. Some studies proposed a significant influence of MSP on the regular/heavy smoking of adult offspring (13, 15), whereas others failed to support such an association (20, 21). In addition, most adolescent smokers are more likely to continue smoking as adults, to become chronic heavy smokers and to have difficulty quitting smoking (22, 23). As the cumulative and substantial effects of smoking are across the life course, it is critical to identify the association of MSP with the development of smoking behaviors in adult offspring.

In the present study, we used data from the UK Biobank to investigate the association between MSP and smoking status, age started smoking, pack years of smoking, and number of unsuccessful stop-smoking attempts in adult offspring. To our knowledge, this study is the first to consider such four smoking behaviors comprehensively.

## 2 Materials and methods

### 2.1 Study population

The UK Biobank is a large, prospective cohort study recruiting more than 500,000 participants aged 37–73 years in 22 assessment centers throughout the UK between 2006 and 2010 (24). Information about exposure to maternal smoking and smoking behaviors were collected at baseline assessment through self-completed touchscreen questionnaires. This study obtained ethical approval from the National Health Services (NHS) National Research Ethics Service (Ref: 16/NW/0274) and all participants provided informed consent.

### 2.2 Assessment of exposure

The information of maternal smoking around birth was reported by their offspring via a touchscreen questionnaire. Participants were asked whether their mothers smoked regularly around the time when they were born with four responses: “Yes,” “No,” “Do not know,” and “Prefer not to answer.” Participants who answered “Do not know” and “Prefer not to answer” were set to missing.

### 2.3 Ascertainment of outcomes

The outcomes in our study included smoking status, age started smoking, pack years of smoking, and number of unsuccessful stop-smoking attempts in adult offspring, all of which were obtained from self-reported questionnaires. Smoking status was collected in three categories: never, previous, or current smoker, which was dichotomized into yes (previous or current) and no. Age started smoking was assessed with the question “How old were you when you first started smoking on most days.” In the UK Biobank, pack years of smoking was defined as the number of cigarettes smoked per day, divided by 20, multiplied by the number of years of smoking. The number of years of smoking was calculated by subtracting the age started smoking from the age stopped smoking (for previous smoker) or age at recruitment (for current smokers). To assess the number of unsuccessful stop-smoking attempts, participants were asked to answer the question” How many times did you try to give up smoking before you were successful.”

### 2.4 Covariates

Potential confounders, reported in the questionnaires, were birth year, sex, ethnicity, Townsend deprivation index, residence, and breastfeeding of the participants, as well as the maternal age of their mothers. Ethnicity was categorized into White, Asian, Black, and others. Townsend deprivation index is a socio-economic measure based on area of residence, of which a higher score implies a higher degree of deprivation.

### 2.5 Statistical analyses

Characteristics of maternal smoking around birth are presented as medians or percentages for continuous and categorical variables, respectively. Additionally, logistic regression was employed to investigate the relationship between maternal smoking and the smoking status of the offspring, which was characterized as a binary outcome. The odds ratio (OR) and 95% confidence interval (95% CI) were used to quantify this association. The relationships between maternal smoking and the age at which offspring began smoking, as well as the number of pack years of smoking, were evaluated using a linear regression model. These outcomes were treated as continuous variables, and the results were reported as beta coefficients with corresponding 95% confidence intervals (CIs). A negative binomial regression model was utilized to investigate the correlation between maternal smoking and the number of unsuccessful stop-smoking attempts made by offspring. The number of attempts was treated as count data, and the results were reported in terms of the incidence rate ratio (IRR) with corresponding 95% confidence intervals (CIs). Adjustments were made for the following potential confounders: birth year, sex, ethnicity, Townsend deprivation index, residence, and breastfeeding. We also conducted subgroup analyses to explore the possible effect modification of sex and breastfeeding. As a sensitivity analysis, we excluded the participants without reporting maternal age and adjusted it in the model. Two-tailed *p* < 0.05 was considered to be statistically significant. All statistical analyses were performed using R (version 4.1.0).

## 3 Results

### 3.1 Characteristics of study population

**Figure 1:**
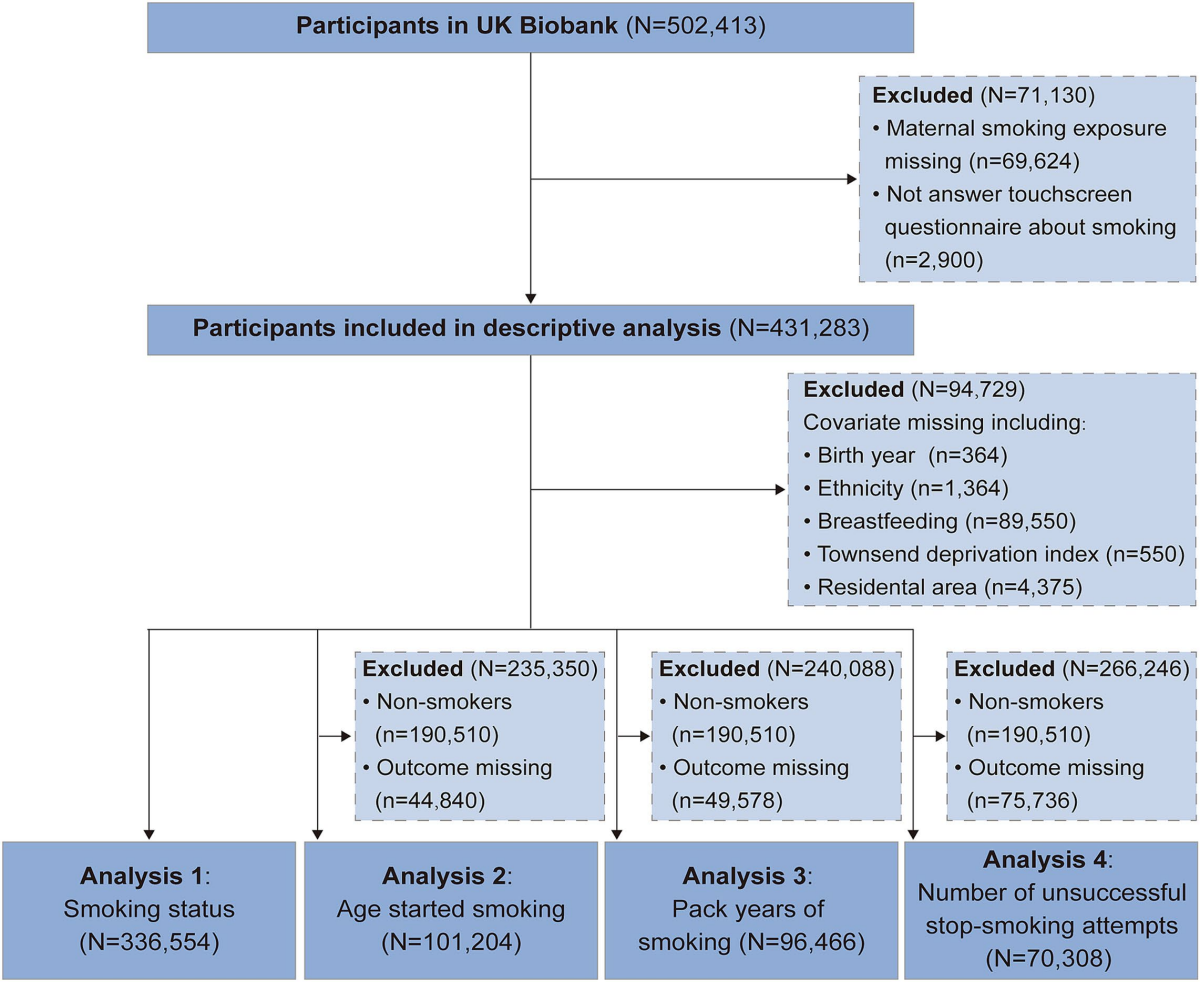
Flowchart of the selection of study population.

Figure 1 shows the participant inclusion and exclusion in this study. Of the 502,413 participants available in the UK Biobank, we excluded participants who did not self-report maternal smoking exposure and touchscreen questionnaire about smoking (*n* = 71,130). Therefore, 431,283 participants were included in the descriptive analysis. Furthermore, after excluding participants with missing data on covariates (*n* = 94,729), we included 156,604 participants in the analysis 1 of smoking status. On the basis of non-smokers and non-missing outcomes, 101,204 participants in the analysis 2 of age started smoking, 96,466 participants in the analysis 3 of pack years of smoking, and 70,308 participants in the analysis 4 of number of unsuccessful stop-smoking attempts. Description of characteristics of study population are shown in Table 1. Of these, 29.27% (*n* = 126,222) exposed to maternal smoking around birth. Among offspring with maternal smoking, 46.68% were male, 98.55% were White, and 86.83% lived in urban areas. Compared to non-exposed offspring, those who reported maternal smoking around birth were more likely to be younger, males, Whites, to have a mother giving birth at a younger age, and to have a lower social-economic status.

**Table 1:** Characteristics of study population based on maternal smoking around birth.

	Overall (*n* = 431,283)	Maternal smoking around birth		Missing, n (%)
		No (*n* = 305,061)	Yes (*n* = 126,222)	
Birth year, median (IQR)	1,951 (1,946, 1,959)	1,951 (1,945, 1,959)	1,952 (1,947, 1,959)	364 (0.08)
Male, n (%)	195,035 (45.22)	136,114 (44.62)	58,921 (46.68)	0 (0)
Ethnicity, n (%)				1,364 (0.32)
White	405,018 (94.21)	280,978 (92.41)	124,040 (98.55)	
Asian	10,944 (2.55)	10,605 (3.49)	339 (0.27)	
Black	7,404 (1.72)	7,117 (2.34)	287 (0.23)	
Others	6,553 (1.52)	5,359 (1.76)	1,194 (0.95)	
Maternal age, median (IQR)	26.81 (4.88)	27.02 (4.89)	26.15 (4.82)	253,935 (58.88)
Breastfeeding, n (%)	248,624 (72.75)	185,642 (75.60)	62,982 (65.49)	89,550 (20.76)
Townsend deprivation index, median (IQR)	−2.17 (−3.66, 0.49)	−2.23 (−3.70, 0.37)	−2.00 (−3.56, 0.79)	550 (0.13)
Urban, n (%)	367,585 (86.10)	259,109 (85.80)	108,476 (86.83)	4,375 (1.01)

IQR, interquartile range.

### 3.2 Association between maternal smoking around birth and smoking behaviors in offspring

**Figure 2:**
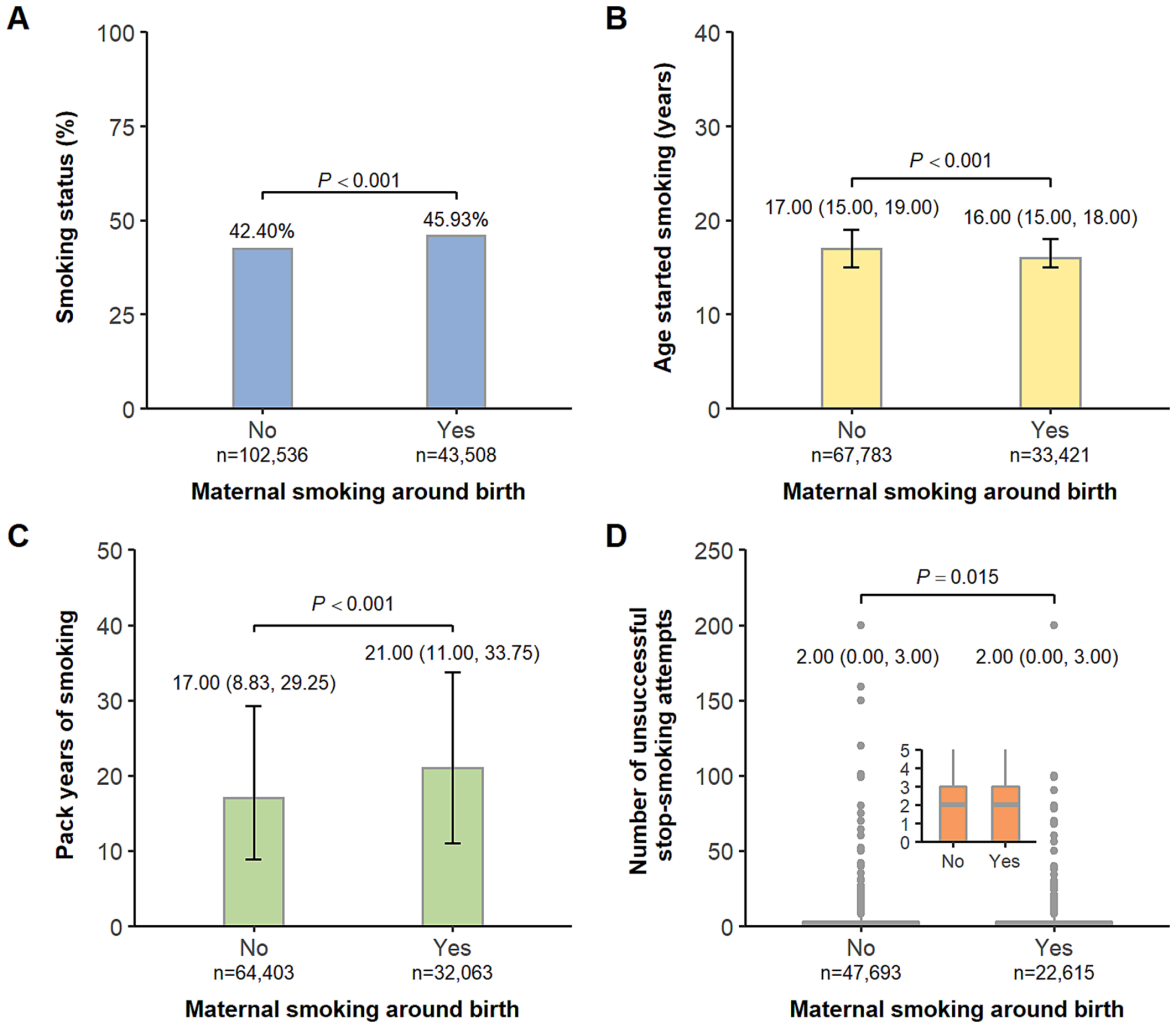
Comparison of offspring’s smoking status **(A)**, age started smoking **(B)**, pack years of smoking **(C)**, and number of unsuccessful stop-smoking attempts **(D)** by maternal smoking around birth. **(A)** Data are presented as percentages. **(B–D)** Box plots show Q1-median-Q3 with range and gray dots are outlier values.

As depicted in Figure 2, we found offspring exposed to maternal smoking around birth were more likely to be either previous or current smokers. Among the offspring who ever smoked, those in the exposed group tended to start smoking at an earlier age and have more pack years of smoking. The associations of maternal smoking around birth with the four measures of offspring smoking behaviors are presented in Table 2. In the crude models, the exposure of interest was associated with all the outcomes in an unfavorable manner (all *p* < 0.001). When adjusted for potential confounders including birth year, sex, ethnicity, Townsend deprivation index, residence, and breastfeeding, the estimates were altered to a different degree but stayed statistically significant (all *p* < 0.001). We observed an 8% (95% CI: 7–10%) increased risk of being smokers in offspring with a history of maternal smoking around birth, as compared to those without. Among the offspring who ever smoked, people in the exposed group started smoking 0.83 (95% CI: 0.77–0.89) years younger, had 3.51 (95% CI: 3.28–3.74) higher pack years of smoking, and experienced 0.11 (95% CI: 0.09–0.13) more times of unsuccessful stop-smoking attempts compared with those in the non-exposed group. In the sensitivity analysis, the associations between maternal smoking around birth and the four measures of offspring smoking behaviors did not materially change (Supplementary Table S1).

**Table 2:** Association between maternal smoking around birth and smoking behaviors in offspring.

Outcome	No. of participants	Crude model		Adjusted model^a^	
		Estimate (95% CI)	*p* value	Estimate (95% CI)	*p* value
Smoking status	336,554	1.15 (1.14, 1.17)	<0.001	1.08 (1.07, 1.10)	<0.001
Age started smoking, year	101,204	−0.92 (−0.98, −0.87)	<0.001	−0.83 (−0.89, −0.77)	<0.001
Pack years of smoking, pack-year	96,466	3.63 (3.39, 3.87)	<0.001	3.51 (3.28, 3.74)	<0.001
Number of unsuccessful stop-smoking attempts	70,308	1.10 (1.08, 1.12)	<0.001	1.11 (1.09, 1.13)	<0.001

Non-smokers was used as a reference group in all models. The estimates indicate odds ratio for smoking status, beta coefficients for age started smoking and pack years of smoking, and incidence rate ratio for number of unsuccessful stop-smoking attempts, respectively.

a Adjusted for birth year, sex, ethnicity, Townsend deprivation index, residence and breastfeeding.

CI, confidence intervals.

### 3.3 Subgroup analyses

**Figure 3:**
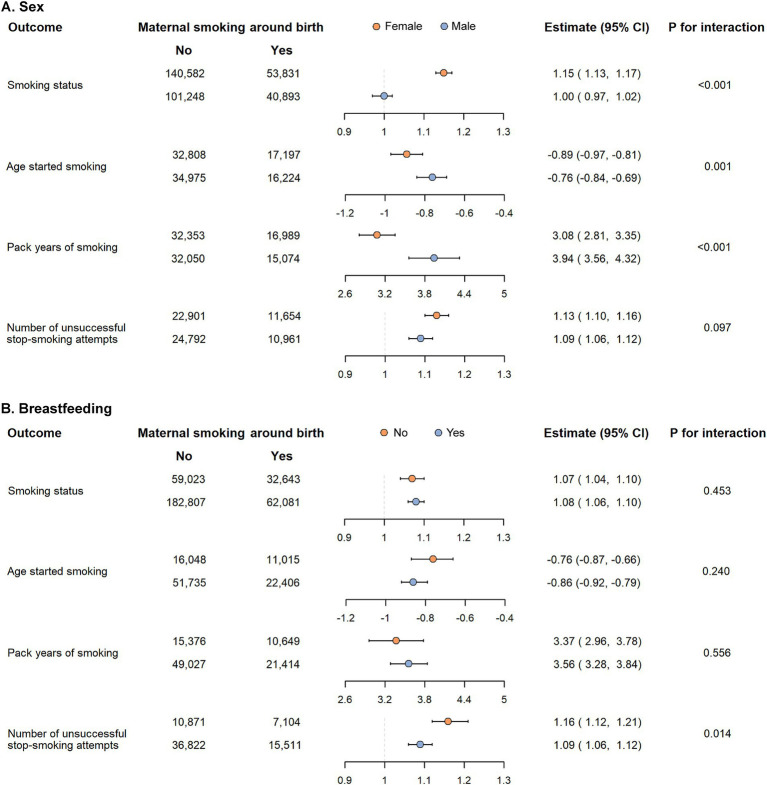
Subgroup analysis of the association between maternal smoking around birth and smoking behaviors in offspring according to sex **(A)** and breastfeeding **(B)**.

In subgroup analyses, when stratified by sex, the excessive risk related to maternal smoking around birth was observed in females for smoking status (OR: 1.15, 95% CI: 1.13–1.17, *P_interaction_* < 0.001) and age started smoking (beta per year: −0.89, 95% CI: −0.97 to −0.81, *P_interaction_* = 0.001) but in males for pack years of smoking (beta per pack-year: 3.94, 95% CI: 3.56–4.32, *P_interaction_* < 0.001, Figure 3A). When stratified by breastfeeding, the excessive risk was observed in the non-breastfeeding group only for number of unsuccessful stop-smoking attempts (IRR: 1.16, 95% CI: 1.12–1.21, *P_interaction_* = 0.014, Figure 3B). However, the positive association between maternal smoking around birth and other smoking behaviors were similar across all subgroups (all *P_interaction_* > 0.05).

## 4 Discussion

In this study, we found exposure to maternal smoking around birth was associated with increased risk of offspring smoking. In smokers, such an exposure was additionally associated with early onset of smoking, high pack years of smoking, and increasing number of unsuccessful stop-smoking attempts. We also observed possible effect modification of sex and breastfeeding on certain smoking behaviors in offspring.

Our results that MSP was associated with an increased risk of offspring’s smoking agree with previous reports (7–9, 13, 15, 25) and extend the finding to a later life. However, we estimated that 8% higher risk of smoking in offspring of smoking mothers, which was lower than other studies (7–9). It was possibly due to the different definition of offspring smoking. The mechanism underlying the association was unclear. One proposed genetic-related explanation might be that tobacco toxicants and nicotine are directly transferred via the placenta and influence the expression of nicotinic acetylcholine receptors in the brain of fetus, resulting in the high propensity of smoking for offspring (26, 27). Another hypothesis suggests that the association is related to specific environmental risk factors. For example, adolescents may smoke by emulating the smoking behavior of their parents (28). A systematic review conducted in 2011 (29) reported that the risk of adolescents’ tobacco smoking increased significantly by smoking by the mother than the father. In contrast, another systematic review conducted in 2016 (30) suggested that the effects of parental smoking on youth smoking-related cognitions may be rather modest.

We found that exposed to maternal smoking around birth predisposed the offspring to early initiation of cigarette use. A longitudinal birth cohort study in South West England showed a positive association between MSP and offspring smoking initiation (12). Another study also presented that MSP was associated with earlier onset of regular smoking in offspring (31). Nonetheless, Rissanen et al. (32) reported offspring exposed to MSP had the similar starting ages of smoking compared to those who were not exposed.

In addition, maternal smoking around birth associated with offspring’s heaviness of smoking and unsuccessful quit attempts by midlife or aging life. Rissanen et al. (32) also showed the positive association between MSP and smoked pack years in offspring by midlife, supporting our results. However, Rydell et al. (33) conducted a matched cohort study in Sweden and presented that there was no difference with regards to previous quit attempts in exposure-discordant siblings. One possible explanation for the opposite results might be that participants in the study by Rydell et al. were 19–27 years old, which were younger than those of our study.

Our study showed that maternal smoking around birth markedly increased the risk of previous or current smoking and younger starting age in female offspring, which was in line with previous studies (19, 20, 34). Similarly, the finding was also observed in the offspring of rats exposed to maternal prenatal tobacco use (35). In contrast, Duko et al. (8) reported no gender differences in the association between exposure of maternal prenatal smoking and offspring smoking, however based on a relatively small sample size (*n* = 1,210). Interestingly, male offspring exposed to MSP had higher pack years of smoking than female offspring. Li et al. (34) detected that genetic factors played a more significant role for smoking initiation but a less significant role for smoking persistence in female adults compared to male adults, which could support our findings. Furthermore, our study indicated an interaction between breastfeeding and MSP on number of unsuccessful stop-smoking attempts in offspring. The offspring without breastfed by smokers had more times of unsuccessful quit-smoking attempts than others. Considering the negative consequences of smoking on child health, mothers smoking heavily during pregnancy prefer not to breastfeed (36), leading their infants to greater intrauterine tobacco exposure in turn. However, this interaction should be interpreted with caution given lack of relevant studies.

To our knowledge, our study is the first to simultaneously assess the associations between maternal smoking around birth and four outcomes of offspring smoking behaviors in middle-aged and older adults. Another strength of this study is the large sample size, ensuring the sufficient statistical power. However, our study has several limitations. First, although various covariates were considered in our analysis, residual confounding, such as postnatal maternal smoking and paternal smoking during the pregnancy and postpartum period, might exist. But several studies suggested that MSP still associated with offspring smoking after adjustment for these covariates (8, 9). Second, lacking the details of maternal smoking around birth at different trimesters or in the postpartum made it difficult to further investigate the possible dose–response relationship. The third limitation is the potential for recall bias due to the use of retrospective questionnaires to collect data on maternal smoking around the time of birth. Previous studies have found that people may not accurately recall their smoking behavior, particularly over longer periods of time (37), thus we admit that recall bias may be a significant concern in our study, as participants may have difficulty accurately recalling their mother’s smoking behavior. While we attempted to minimize the impact of recall bias by excluding participants who reported being uncertain about their mother’s smoking status, it is possible that some level of bias remains. Future studies could consider using prospective data, such as cotinine levels (38), to validate the accuracy of parental recall of smoking behavior. Fourth, some variables are not provided by UK Biobank, thus were not included in the statistical models such as family substance disorders, addictive disorders, alcohol use disorders, mental health issues, social class, peer pressure during adolescence, income, etc. It is possible that these unmeasured confounding factors could have influenced the observed associations between the exposure and outcome variables. Future studies should aim to include a wider range of potential confounders to further investigate the mechanisms underlying this association. Lastly, generalizing the results of our study to the general population should be cautious because the participants in the UK Biobank were volunteers and dominantly of European ancestry. Additionally, it is crucial to replicate this study in groups with different racial and socioeconomic backgrounds. Finally, the findings of this study are not only important for individual-level interventions but also provide valuable insights for the development of public health policies and group-level interventions. First, public health education can raise awareness of the dangers of smoking during pregnancy and provide more resources and support for smokers. Second, implementing stricter smoking bans to reduce exposure to smoking for pregnant women and children is crucial. Enforcing smoking bans in schools, workplaces, and communities can help reduce the intergenerational transmission of smoking behaviors.

## 5 Conclusion

The findings of our study underline that offspring exposed to maternal smoking around birth had the increased risks of smoking, early onset of smoking, pack years of smoking and number of unsuccessful stop-smoking attempts and the adverse effects might reach at least up to their middle even older age. Smoking cessation before pregnancy should be encouraged to prevent the transmission to the next generation.

## Data Availability

The original contributions presented in the study are included in the article/Supplementary material, further inquiries can be directed to the corresponding authors.
