# Supplementary material for: Association between maternal smoking during pregnancy and smoking behaviors in adult offspring
Source: Front Public Health. 2025 Mar 28;13:1505418. doi: 10.3389/fpubh.2025.1505418 (PMC11985817; doi:10.3389/fpubh.2025.1505418)
Supplement: Supplementary file 1 [file Table_1.docx]

**Table S1. Sensitivity analysis.**

| **Outcome** | **No. of participants** | **Crude Model** | | **Adjusted Model^a^** | |
| --- | --- | --- | --- | --- | --- |
|  |  | **Estimate (95% CI)** | ***P* value** | **Estimate (95% CI)** | ***P* value** |
| Smoking status | 156,604 | 1.15 (1.13, 1.18) | <0.001 | 1.09 (1.06, 1.12) | <0.001 |
| Age started smoking, year | 42,076 | -0.97 (-1.06, -0.88) | <0.001 | -0.86 (-0.95, -0.76) | <0.001 |
| Pack years of smoking, pack-year | 40,575 | 2.75 (2.43, 3.08) | <0.001 | 2.56 (2.25, 2.88) | <0.001 |
| Number of unsuccessful stop-smoking attempts | 27,812 | 1.07 (1.04, 1.11) | <0.001 | 1.09 (1.05, 1.13) | <0.001 |

Non-smokers was used as a reference group in all models. The estimates indicate odds ratio for smoking status, beta coefficients for age started smoking and pack years of smoking, and incidence rate ratio for number of unsuccessful stop-smoking attempts, respectively.

^a^Adjusted for birth year, sex, ethnicity, maternal age, Townsend deprivation index, residence and breastfeeding.

CI, confidence intervals.
